# Nanocavity crossbar arrays for parallel electrochemical sensing on a chip

**DOI:** 10.3762/bjnano.5.124

**Published:** 2014-07-23

**Authors:** Enno Kätelhön, Dirk Mayer, Marko Banzet, Andreas Offenhäusser, Bernhard Wolfrum

**Affiliations:** 1 Institute of Bioelectronics (PGI-8/ICS-8) and JARA-Fundamentals of Future Information Technology, Forschungszentrum Jülich, 52425 Jülich, Germany. Current address: Department of Chemistry, Physical and Theoretical Chemistry Laboratory, Oxford University, South Parks Road, Oxford, OX1 3QZ, United Kingdom; 2 Institute of Bioelectronics (PGI-8/ICS-8) and JARA-Fundamentals of Future Information Technology, Forschungszentrum Jülich, 52425 Jülich, Germany; 3 Institute of Physics, RWTH Aachen University, 52074 Aachen, Germany

**Keywords:** electrochemical imaging, nanoelectrochemistry, redox cycling

## Abstract

We introduce a novel device for the mapping of redox-active compounds at high spatial resolution based on a crossbar electrode architecture. The sensor array is formed by two sets of 16 parallel band electrodes that are arranged perpendicular to each other on the wafer surface. At each intersection, the crossing bars are separated by a ca. 65 nm high nanocavity, which is stabilized by the surrounding passivation layer. During operation, perpendicular bar electrodes are biased to potentials above and below the redox potential of species under investigation, thus, enabling repeated subsequent reactions at the two electrodes. By this means, a redox cycling current is formed across the gap that can be measured externally. As the nanocavity devices feature a very high current amplification in redox cycling mode, individual sensing spots can be addressed in parallel, enabling high-throughput electrochemical imaging. This paper introduces the design of the device, discusses the fabrication process and demonstrates its capabilities in sequential and parallel data acquisition mode by using a hexacyanoferrate probe.

## Introduction

Redox cycling represents a powerful method for the detection of analytes that can participate in repeated redox reactions [1–8]. Sensors typically use two electrodes that are located in close proximity to each other and can be biased individually. During operation one electrode is set to a potential above the redox potential *E*_0_ of the analyte under investigation, while the other electrode is set below this potential. Molecules can repeatedly participate in subsequent redox reactions at the electrodes, hence forming a current across the gap. This current can then be measured externally and allows one to draw conclusions regarding a variety of analytes or reaction characteristics such as the electrode kinetics or the analyte concentration.

A distinct advantage of this technique over conventional amperometry, using a single working electrode, is given by the increased Faradaic current caused by the redox cycling effect. A single molecule entering the sensor does not only contribute with an individual charge transfer to the Faradaic current but participates in multiple reactions that each result in a charge transfer to the working electrode. Sensitivity differs among sensor designs and is mainly determined by the collection efficiency of the sensor and the average time a molecule requires for passing one redox cycle. In recent years, a variety of on-chip redox cycling devices has been implemented. Hereby, the highest sensitivity per electrode area was reported for nanofluidic redox cycling sensors. These sensors feature micron-sized electrodes that are arranged in parallel to each other and the wafer surface, being separated by a nano-scaled gap [9–11]. The current per molecule obtained with such a sensor featuring the inter electrode distance *h* is directly proportional to *h*^−2^. Therefore, small inter-electrode distances can significantly amplify the electrochemical signal [12–13]. Amplification factors can be calculated via comparison to a single electrode of the same size and may reach several orders of magnitudes, allowing very low detection limits; Recently, Lemay’s group reported the ultimate detection limit by sensing at molecular resolution inside a nanofluidic redox cycling device [14–16]. Besides the advantages of electrochemical amplification, redox cycling sensors allow for the formation of large, dense arrays of electrochemical sensors that are highly desirable for applications such as on-chip parallel biosensing or the detection of chemical communication in a neuronal network. This can be achieved via the organization of feed lines in a perpendicular arrangement. Individual sensors are then located at each of the feed line intersections. Redox cycling is enabled at the intersection by setting the potentials of two orthogonal feed lines to values above and below the redox potential of an analyte. Even though faradaic currents may also occur at all other electrodes that are connected to the biased feed lines and are exposed to redox-active molecules, their individual contribution to the overall measured current is comparably small due to the strong amplification by the redox cycling effect. Hence, individual sensors can be easily read out by this method.

Addressable redox cycling electrode arrays have been pioneered by the group of Matsue since 2008 and various designs have been reported since then. Implementations include systems, which consist of two wafers of parallel bar electrodes that are glued face-to-face to each other [17–18], arrays of ring–ring-based sensors with orthogonal feed lines [19], and designs featuring interdigitated electrodes at the intersections [20]. Reported applications include gene-function analysis [18], electroluminescence detection [21], mapping of cell topographies [22], detection of cellular enzyme secretion [19,23], detection of DNA hybridization [24], and evaluation of embryoid bodies [25].

This paper describes the design and fabrication of a crossbar-based nanocavity redox cycling sensor array that combines the advantages of the two approaches: crossbar architecture and nanocavity sensors. The large redox cycling amplification of the nanocavity sensors allows such arrays to be operated in a parallel readout for high-throughput applications. The redox cycling response during electrochemical imaging using parallel data acquisition is demonstrated and different modes of operation for its future use in mapping neurochemical events in cell culture are discussed.

## Results and Discussion

**Figure 1:**
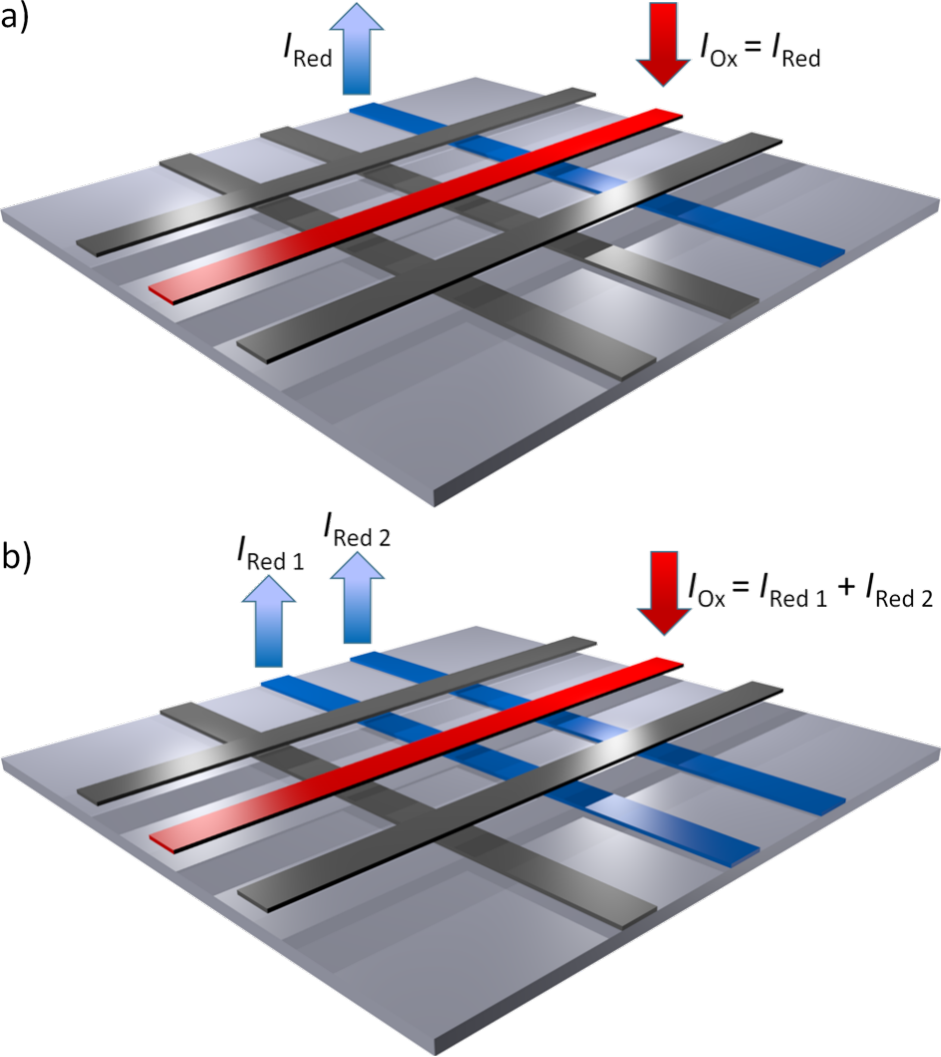
Illustration of the two modes of operation: a) Sequential data acquisition: Each intersection is read out sequentially. b) Parallel data acquisition: Intersections are read out simultaneously in a row-wise fashion.

Our sensors offer two different modes of operation: The sequential and the parallel readout mode. During sequential data acquisition, each crossing point on the sensor is addressed individually one after the other, while the electrochemical image of the sensor surface is assembled afterwards. As it can be seen in Figure 1a, two electrodes that are arranged perpendicular to each other are set to potentials above and below the redox potential, while all other electrodes remain unbiased. Redox cycling is then enabled at the corresponding intersection and the respective redox-cycling current can be detected at both electrodes. In parallel acquisition mode, however, two or more electrodes that are arranged perpendicular to the one oxidizing electrode are biased to reducing potentials. Hence, redox cycling is enabled at each intersection between a reducing electrode and the oxidizing electrode, thus, resulting in a row-wise read-out. As sketched in Figure 1b, the individual redox-cycling currents of each intersection can be measured at the reducing electrodes, while the current at the oxidizing electrode amounts to the sum of all other redox cycling currents.

**Figure 2:**
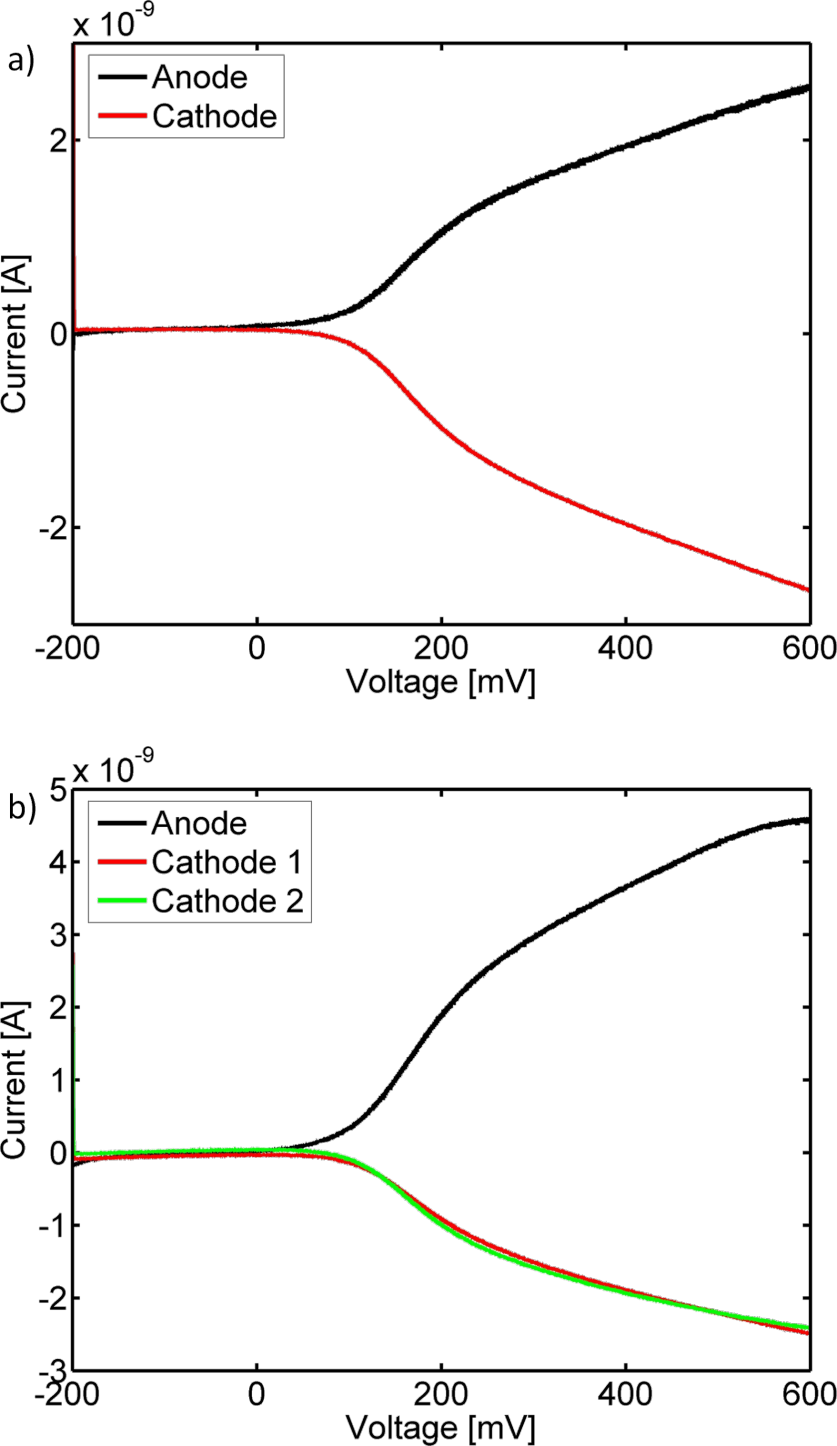
Redox cycling currents: a) Cyclic voltammogram detected in sequential data acquisition mode at a single intersection. b) Cyclic voltammograms recorded at two intersections during parallel acquisition. Both data sets were recorded in 1 mM potassium hexacyanoferrate in PBS and filtered via a Butterworth filter. Furthermore, the data were offset corrected and traces recorded in a single electrode setup were subtracted from the anodic currents in order to isolate the respective redox cycling currents.

Figure 2 compares data of the sensor during operation in both acquisition modes in presence of 1 mM hexacyanoferrate. Hereby, both graphs exhibit some characteristics in common: Below the redox potential, which can be found around 180 mV, the current does not increase with the anodic voltage. However, above the redox potential there is a fast increase in current, which is due to the now enabled redox cycling. Nevertheless, the current does not reach the expected diffusion-limited steady state at high overpotentials. This effect can be attributed to kinetic limitations that may be caused by impurities on the electrode surfaces that remain from the fabrication process. Furthermore, in both cases the anodic and the sum of the cathodic currents equal. Thus, redox cycling inside the sensor is highly efficient.

**Figure 3:**
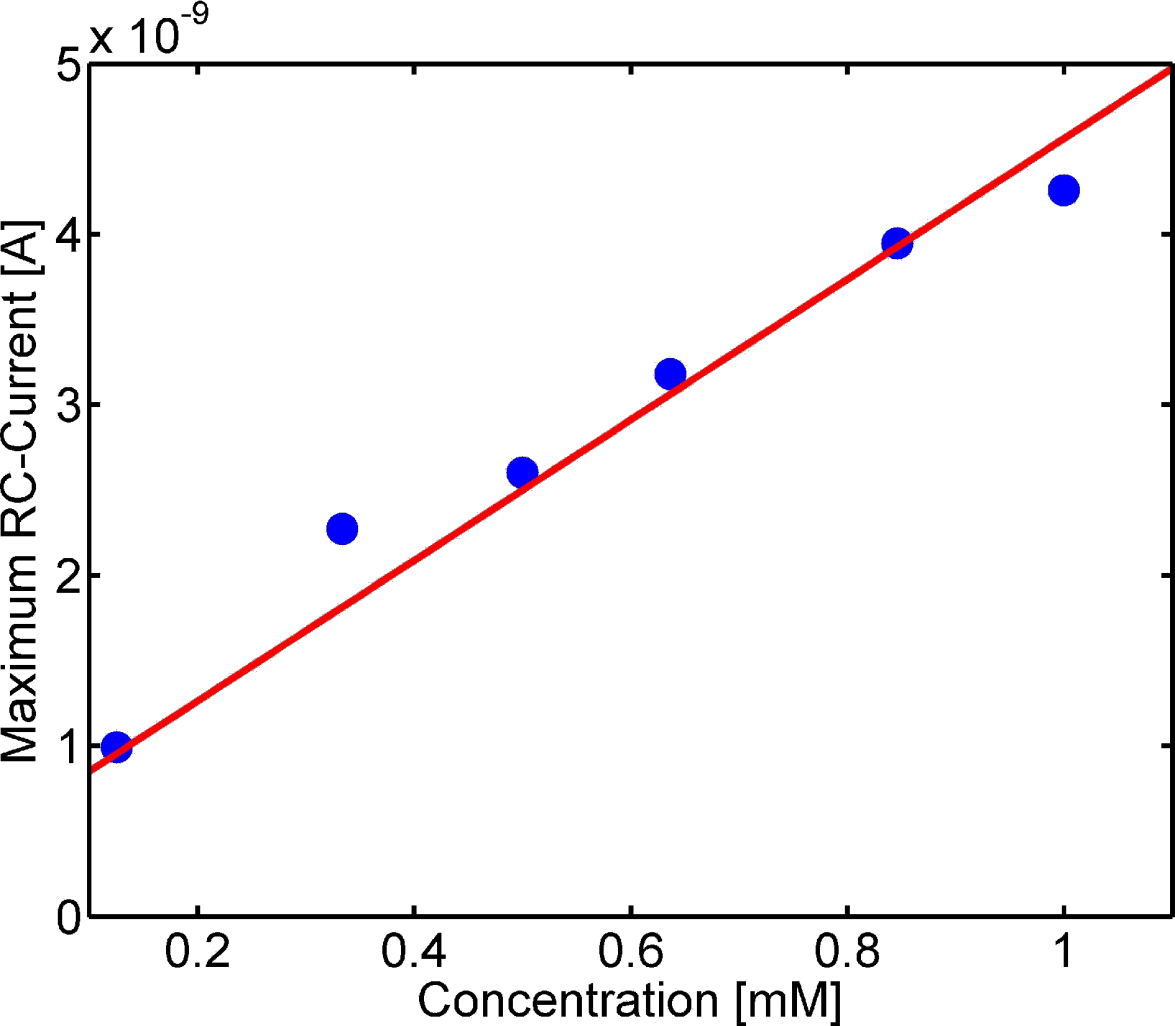
Concentration dependency of an intersection in sequential data acquisition. Data was recorded during cyclic voltammograms at different concentrations of potassium hexacyanoferrate in 100 mM KCl and represents the absolute difference between the cathodic peak current and a measurement using only the background electrolyte.

**Figure 4:**
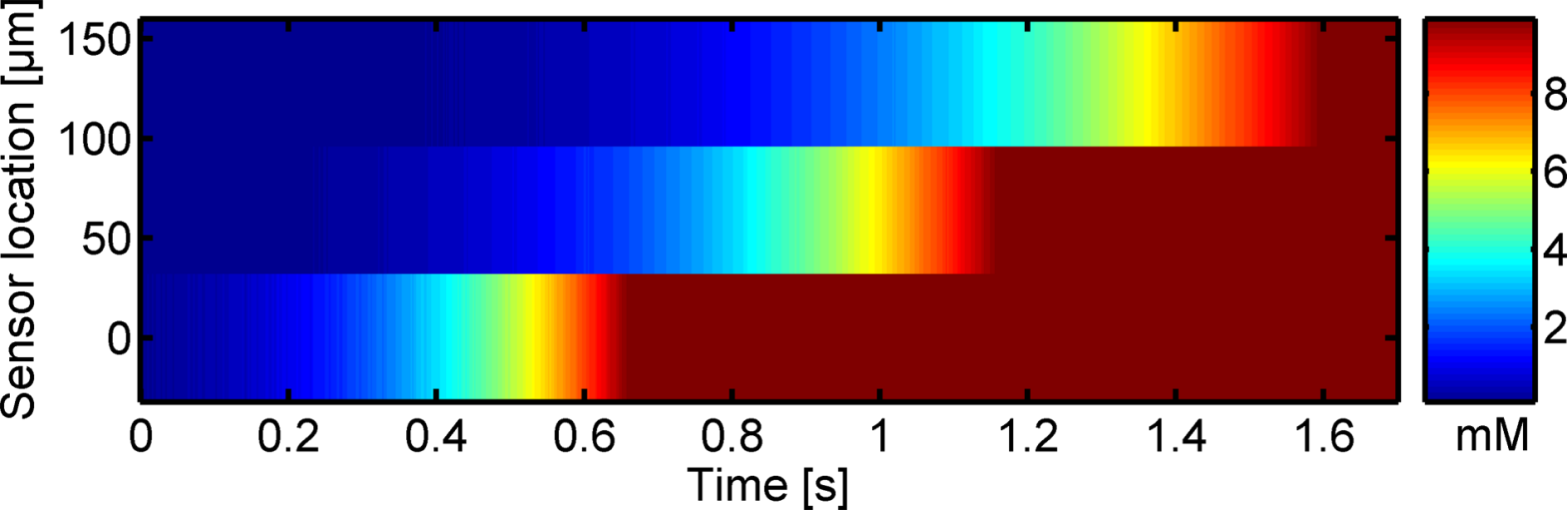
Electrochemical recording of the change in concentration during dissolution of a potassium haxacyanoferrate crystal above the sensor surface.

Figure 3 demonstrates the concentration dependency of an individual sensor. Hereby, a single intersection was characterized at varied concentrations of potassium hexacyanoferrate in sequential acquisition mode. As it can be seen in the plot the redox cycling current scales approximately linearly with the concentration. The slope of the sensor response was obtained via a linear regression yielding a sensitivity of 2.4 ± 0.2 × 10^4^ A·m^−2^·M^−1^ in the overlapping electrode area (1.68 × 10^−12^·m^2^). Figure 4 shows a typical sensor response but the array exhibits a large variance in sensitivity making it necessary to calibrate individual sensors for quantitative imaging. The largest current responses obtained were in the range of 1.7 × 10^5^ A·m^−2^·M^−1^, which is still significantly less than the theoretically expected diffusion-limited value for the devices in case of a one-electron process if we neglect kinetic limitations

[1]
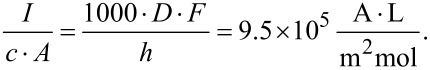


Here, *c* is the concentration in (mol/L), *A* the overlapping electrode area in m^2^, *D* = 0.64 × 10^−9^ m^2^/s the diffusion coefficient, *F* = 96485 C/mol the Faraday constant, and *h* = 65 nm the nanocavity height. Deviations between expected and recorded current responses in nanocavity devices have been observed previously and are thought to depend on fabrication inhomogeneities and residues as well as adsorption effects limiting the electrode kinetics.

In order to demonstrate parallel electrochemical recording at the nanocavity crossbar array, we monitor concentration fluctuations at the chip surface induced by the dissolution of a hexacyanoferrate crystal [26]. For this purpose, all but one electrode are biased to a reducing potential of −200 mV, while the one electrode is biased to an oxidizing potential of 600 mV. In this operation mode, redox cycling is simultaneously enabled at all 16 sensors along the oxidizing electrode and the respective sensor signal can be read out at the corresponding perpendicular cathodes. The device is then calibrated in plain 100 mM KCl solution as well as in a 1 mM potassium hexacyanoferrate solution in order to linearly interpolate the response curve for the individual sensors. After adding the 1 mM potassium hexacyanoferrate solution, three sensors that are located next to each other (the yield of functional sensors is approximately 40%) are chosen and a potassium hexacyanoferrate crystal is added to the solution. The so obtained electrochemical image of its dissolution after background subtraction can be seen in Figure 4. The parallel readout of the crossbar array allows the chemical concentration to be mapped at all active sensors simultaneously with a millisecond temporal resolution, which is sufficient for resolving fast dissolution processes. The sensitivity is determined by the height of the nanocavities (65 nm) while the spatial resolution relies on the sensor array density (ca. 244/mm^2^). When using the current fabrication process, the sensor density is limited by the low yield of functional intersections, which is probably related to stability issues with the passivation layer. Solving this problem would in principle allow for high-density mapping in the range of 10000/mm^2^ for reasonable cross bar pitches of 10 μm as obtainable by conventional optical lithography.

## Conclusion

We introduced the design and fabrication of a novel device for the electrochemical on-chip imaging of redox molecules by redox cycling. The presented chip was fabricated with standard cleanroom technology and features nanocavity redox cycling devices in a crossbar architecture for sensitive electrochemical detection at a high sensor density. Measurements in potassium hexacyanoferrate solution are shown and different modes of operation are demonstrated: the sequential readout of individual sensors and the parallel readout mode, which allows for the spatiotemporal sensing along one feed line.

**Figure 5:**
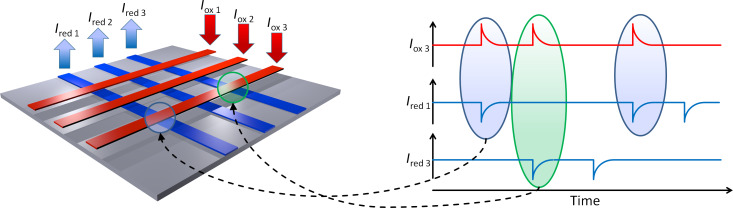
Illustration of a future electrochemical setup for parallel spike recording on-chip. By correlation of individual spike events at anodic and cathodic electrodes, all electrodes intersections can be read out simultaneously.

It can be assumed that the presented technique, which combines high-density sensing of electrochemical species with redox cycling amplification in the nanofluidic cavities, will be advantageous for electrochemical imaging methods and electrochemical biological assays. Particularly, one may expect that the detection or mapping of neutrotransmitter secretion (such as the redox-active molecule dopamine [13]) in neuronal networks will be one of the most interesting applications [27–37]. In this case, the sensor array is exposed to fast fluctuations in the neurotransmitter concentration. By biasing the two parallel sets of bar-electrodes to reducing and oxidizing potentials, one can then correlate the electrochemical signals at orthogonal electrodes, hence recording data from all sensors simultaneously (see Figure 5). Overall, we expect a wide range of applications for high-density nanocavity sensors and remain looking forward to see their implementation in future imaging systems.

## Experimental

### Sensor design

**Figure 6:**
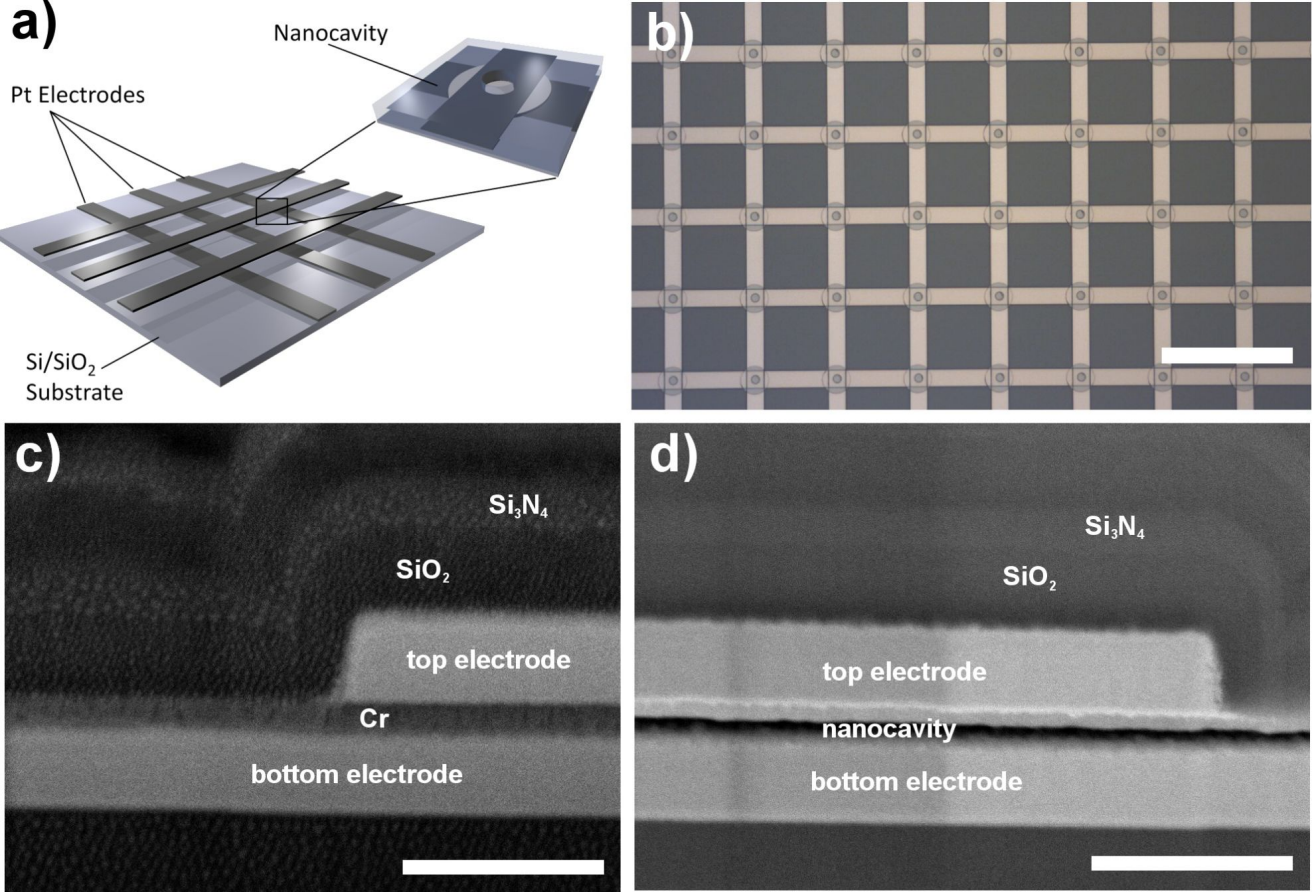
Nanocavity array chip: a) Illustration of a sensor array. The inset sketches a nanocavity sensor that can be found at each feed-line intersection. The light grey spherical layer represents the nanocavity, while the hole in the top electrode and in the passivation layer is the access channel that connects the bulk reservoir on the chip surface to the nanocavity. b) Microscopic top view of a part of the array. c,d) Scanning electrochemical microscope images of FIB-induced cross sections of a nanocavity sensor before (c) and after (d) removal of the sacrificial chromium layer. The scale bars for the images in (b), (c), and (d) are 100 μm, 400 nm, and 400 nm, respectively.

Our device features two orthogonal sets of 16 parallel bar electrodes, each. These electrodes are 14 μm wide, separated by 64 μm (center to center), and fabricated in parallel to the wafer surface. At each intersection, the electrodes are separated by an about 65 nm wide gap, while the arrangement is stabilized by a thick passivation layer that covers the whole device. The inter-electrode area is connected to the bulk reservoir via small access channels that interpenetrate the passivation layer and enable diffusive access to a bulk reservoir. An illustration of the sensor array and a top view microscopic image as well as cross sections of the nanocavity sensor, cut by a focused ion beam (FIB), can be found in Figure 6.

### Fabrication

Devices are structured by means of optical lithography and are processed in class-100 cleanroom facilities. Nanocavities at the intersections between platinum electrodes are formed via the deposition of a sacrificial layer followed by an isotropic etch. The full device is stabilized by a silicon oxide/silicon nitride stack that covers the full device and is solely opened trough access holes above each crossbar intersection. Since the whole device is covered by the passivating layer, electrodes can only be accessed from within cavity, while a connection to the bulk reservoir is only enabled through the access channels.

The sensor is fabricated on a thermally oxidized silicon substrate while all structures are formed via lift-off processes or reactive ion etching. Electrodes are fabricated by depositing a titanium/platinum/chromium stack that features the thicknesses 7/50/7 nm by using electron beam evaporation. In the next step, 50 nm thick chromium sacrificial layers are deposited at the positions of the future intersections. These layers define the geometric features of the nanocavities. Afterwards, the top electrodes are fabricated from an electron-beam evaporated stack of chromium/platinum/titanium stack of the thicknesses 7/50/7 nm. Subsequently, a passivation composed of alternating layers. SiO_2_/Si_3_N_4_/SiO_2_ is deposited via plasma enhanced chemical vapor deposition [38]. In the next step, access holes are etched through the passivation directly down onto the chromium sacrificial layer by reactive ion etching. The chromium is then fully removed in an isotropic wet etch using chrome etch solution.

### Electrochemical methods

Electrochemical characterization is either performed via cyclic voltammetry or amperometry. Cyclic voltammograms were recorded using an EPC 10 patch clamp system (HEKA Elektronik Dr. Schulze GmbH, Lambrecht, Germany) and the corresponding software Patch Master. Hereby, one bar electrode is swept from −200 mV to 600 mV and then sweept reverse from 600 mV to −200 mV at a rate of 80 mV/s, while the corresponding other electrode(s) remain at a reducing potential of −200 mV. Parallel redox cycling amperometric measurements for electrochemical imaging are performed by using a custom-built amplifier system (picoAmp64) [36]. The electrodes are set to constant potentials of either −200 mV or 600 mV. All measurements are performed after a equilibration time of 10 s, while the potential of the solution is controlled through an Ag/AgCl reference electrode.
